# Theta Rhythm‐Based Attention Switch Training Effectively Modified Negative Attentional Bias

**DOI:** 10.1111/cns.70157

**Published:** 2024-12-20

**Authors:** Guo Li, Xueli Cai, Yifeng Wang

**Affiliations:** ^1^ Institute of Brain and Psychological Sciences, Sichuan Normal University Chengdu China; ^2^ School of Psychology, South China Normal University Guangzhou China; ^3^ Psychological Research and Counseling Center Southwest Jiaotong University Chengdu China

**Keywords:** attentional bias, attentional bias modification training, cognitive training, depression, theta rhythm

## Abstract

**Background:**

Attentional bias modification training (ABMT) is commonly employed to regulate negative attentional bias (NAB) and, in turn, to prevent or alleviate depressive symptoms. Recent advancements in attention switch theory have facilitated the development of a novel training paradigm that may enhance the efficacy of such interventions.

**Methods:**

A total of fifty‐seven college students were assigned to two groups: one exhibiting NAB and the other without. Both groups underwent training with a novel paradigm integrating theta rhythm with the traditional dot‐probe task (DPT). The DPT was also administered as a pre‐ and post‐test measure.

**Results:**

For individuals with NAB, rhythmic DPT effectively alleviates their NAB. Additionally, within the training procedure's DPT, flashing negative stimuli elicits faster responses when the probe appears at the positive stimulus' location. Baseline attention scores can negatively predict changes in subsequent corresponding attentional performance.

**Conclusions:**

This study presents a novel training paradigm—the theta rhythm‐based DPT—that effectively modifies NAB. The mechanism underlying this intervention may be driven by positive salient stimuli at the critical trough, facilitating the switch of attention from negative to positive stimuli.

## Graphical abstract

This study provided a novel training paradigm, the theta rhythm‐based dot probe task, to effectively modify individuals' negative attentional bias. The underlying mechanism of this training may be to facilitate the switch of attention from negative stimuli to positive ones.

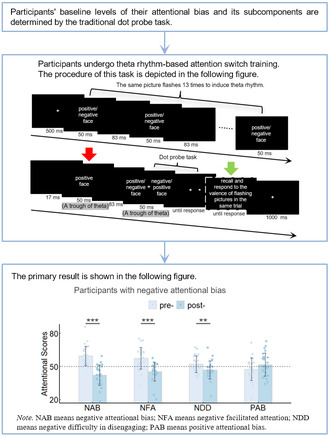

## 1 Introduction

Major depressive disorder (MDD) is the most prevalent mental health condition, characterized by persistent low mood, reduced energy and interest, impaired sleep, and an increased risk of suicidal behavior [1]. It affects over 350 million people worldwide and is the leading cause of disability, contributing significantly to the global burden of disease [2]. Negative attentional bias (NAB), defined as the tendency to allocate more attentional resources to negative information in the environment [3], is a core symptom in individuals with MDD. According to the components theory, NAB consists of two primary components: facilitated attention and difficulty in disengagement [4]. Facilitated attention refers to the tendency to allocate attention more easily or rapidly to negative stimuli, while difficulty in disengagement refers to the challenge of shifting attention away from negative stimuli once it has been captured [5, 6]. Excessive attention to negative stimuli may contribute to the onset, persistence, and recurrence of depression [7, 8]. Consequently, modifying NAB is thought to be a potential strategy for alleviating or preventing depressive symptoms.

A systematic approach for modifying attentional bias (AB) is attentional bias modification training (ABMT). The most commonly used paradigm for this training is the dot probe task (DPT) [9]. The core logic of this approach is that the likelihood of an association between the target and non‐negative stimuli is considerably higher than the probability of an association with negative stimuli, thereby encouraging trainees to direct more attention to non‐negative stimuli. Evidence indicates that individuals predisposed to depression, those clinically diagnosed with depression, individuals with residual depressive symptoms, and other groups associated with depression can, to varying extents, modify their NABs or alleviate depressive symptoms following ABMT. However, the effects of the training are not always consistent [10, 11, 12, 13, 14]. These inconsistent outcomes may stem from various factors, with theoretical limitations being a key contributing cause. For instance, changes in probability may prompt individuals to adjust their criteria for evaluating positive and negative stimuli, rather than altering their attentional preferences toward these stimuli [15].

Fiebelkorn and Kastner [16] have recently proposed a rhythmic theory of attention, which states that theta rhythm organizes attentional states into alternating periods of perceptual sensitivity. Specifically, at the peak of the theta wave, heightened sensory processing and inhibition of eye movements further enhance attentional performance. In contrast, at the trough of the theta wave, sensory processing diminishes and movement inhibition is released, increasing the likelihood of attentional shifts. According to this theory, presenting a stimulus during the trough of the theta wave may be particularly effective in directing attention toward that stimulus.

Presenting a stimulus at a fixed frequency can drive neural activity to oscillate at the same frequency, aligning with the phase of the input stimulus [17, 18]. Currently, neural entrainment is the primary method for modulating neural rhythms through cognitive tasks [5, 6, 19]. For example, auditory stimuli presented at 10 Hz can enhance neural oscillations in the alpha band, thereby improving attentional allocation [20]. Attention networks can also be modulated by rhythmic tasks at lower frequencies, such as 0.1 Hz or even 0.05 Hz [21, 22].

Given the positive outcomes from studies on rhythmic attention regulation, we sought to determine whether rhythm‐based techniques could serve as a novel approach for modifying AB. Based on the rhythmic theory of attention, we introduced a theta rhythm‐based DPT to explore this possibility. First, we employed rhythmic (positive or negative) stimuli to evoke theta oscillations. Second, a recall task was used to direct participants' attention to the rhythmic stimuli, effectively aligning these stimuli with the peak of the theta wave. Third, we presented a positive stimulus at the critical trough of the theta wave to encourage participants to shift their attention toward these stimuli.

## 2 Methods

### 2.1 Study Design

The present study is a 2 (time: pre‐/post‐training) × 2 (group: with/without NAB) mixed design. The group is a between‐subject variable, and time is a within‐subject variable. The study was performed in line with the principles of the Declaration of Helsinki. Approval was granted by the Ethics Committee of the Institute of Brain and Psychological Sciences, Sichuan Normal University (SCNU‐20210105).

### 2.2 Participants

Participants were undergraduate or graduate students recruited from college campuses through social media advertisements. The sample size was determined using G*Power 3.1. The power analysis (effect size *f* = 0.25; power = 0.95; two groups; correlation among repeated measures *r* = 0.50) indicated that a minimum sample size of 54 participants was required. The inclusion criteria were participants aged 18 years or older, with self‐reported absence of any psychological disorders. In total, 25 participants without NAB and 32 participants with NAB were recruited to complete the entire experiment. As an incentive, each participant received a monetary bonus of 30 CNY for their involvement.

### 2.3 Procedure

Participants first completed the *Patient Health Questionnaire* (PHQ‐9), followed by the traditional DPT to obtain baseline scores for AB, facilitated attention, and difficulty in disengagement. Subsequently, the rhythmic training task was administered, followed by a post‐training assessment using the same DPT. Each participant completed all of the aforementioned tasks within 2 days.

### 2.4 Assessments

#### 2.4.1 Dot‐Probe Task

Four emotional face pairings were used in the DPT: sad‐neutral, neutral‐happy, sad‐happy, and neutral‐neutral. The face images were selected from the Chinese Affective Pictures System (CAPS) [23]. Each emotional valence consisted of 24 images, including 12 male and 12 female faces. Within each stimulus pair, faces of the same sex were presented. Table 1 summarizes the statistical differences between the stimulus materials.

**TABLE 1:** Dimensional characteristics of stimuli pairs in the assessment and training procedures.

		Sad	Neutral	Happy	Statistic index
		*M* (SD)	*M* (SD)	*M* (SD)	
Assessment procedure	Valance	3.03 (0.55)	4.67 (0.54)	6.48 (0.66)	*F* (2,69) = 208.11***
	Arousal	5.54 (1.34)	3.58 (0.88)	6.07 (1.10)	*F* (2,69) = 32.86***
Training procedure	Valance	2.96 (0.53)	—	6.91 (0.52)	*t* (30) = 21.46***
	Arousal	5.56 (1.47)	—	6.21 (1.06)	*t* (30) = 1.42

*Note:* *p < 0.05, **p < 0.01, ***p < 0.001.

Abbreviations: M, mean; SD, standard deviation.

**FIGURE 1:**
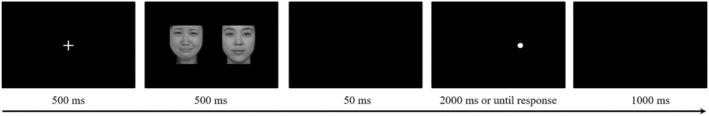
A snapshot of an example from the assessment. The dot probe was randomly displayed following either of the two photos.

At the beginning of each trial, a fixation cross (“+”) was presented for 500 ms, followed by a pair of emotional faces randomly placed on the left and right sides of the screen for 500 ms. A blank screen was then shown for 50 ms, after which a probe dot (“●”) appeared at one of the two previously presented positions with equal probability. Participants were instructed to press the “F” key if the probe dot appeared on the left side and the “J” key if it appeared on the right, responding as quickly and accurately as possible. After the participant's response or if no response was made within 2000 ms, a blank screen was displayed for 1000 ms before the next trial began (see Figure 1). This task consisted of 192 trials, with each of the four stimulus pairs (sad‐neutral, neutral‐happy, sad‐happy, and neutral‐neutral) presented 12 times. Before the formal assessment, participants completed 12 practice trials to familiarize themselves with the procedure.

#### 2.4.2 Self‐Report Measures of Symptoms

The *patient health questionnaire* (PHQ‐9) is a nine‐item assessment tool used to evaluate the severity of depressive symptoms on a four‐point scale, ranging from 0 (not at all) to 3 (almost every day). The total score ranges from 0 to 27, with scores of 0–5 indicating no depression, 6–9 reflecting mild depression, 10–14 indicating moderate depression, 15–19 suggesting severe depression, and 20–27 indicating very severe depression. Before training, participants were asked to complete the questionnaire to assess the presence of each symptom over the past 2 weeks.

### 2.5 Interventions

#### 2.5.1 Rhythmic Training Task

The new paradigm combined the traditional DPT with theta rhythm to train participants to stay away from negative stimuli. The task was programmed by the E‐Prime 2.0 software. The program employed a total of 32 images, comprising 16 happy faces and 16 sad faces, with an equal number of male and female faces. Due to the limited number of sad images, five images of each gender were reused in both the assessment task and the training task. For further information, please refer to Table 1.

**FIGURE 2:**
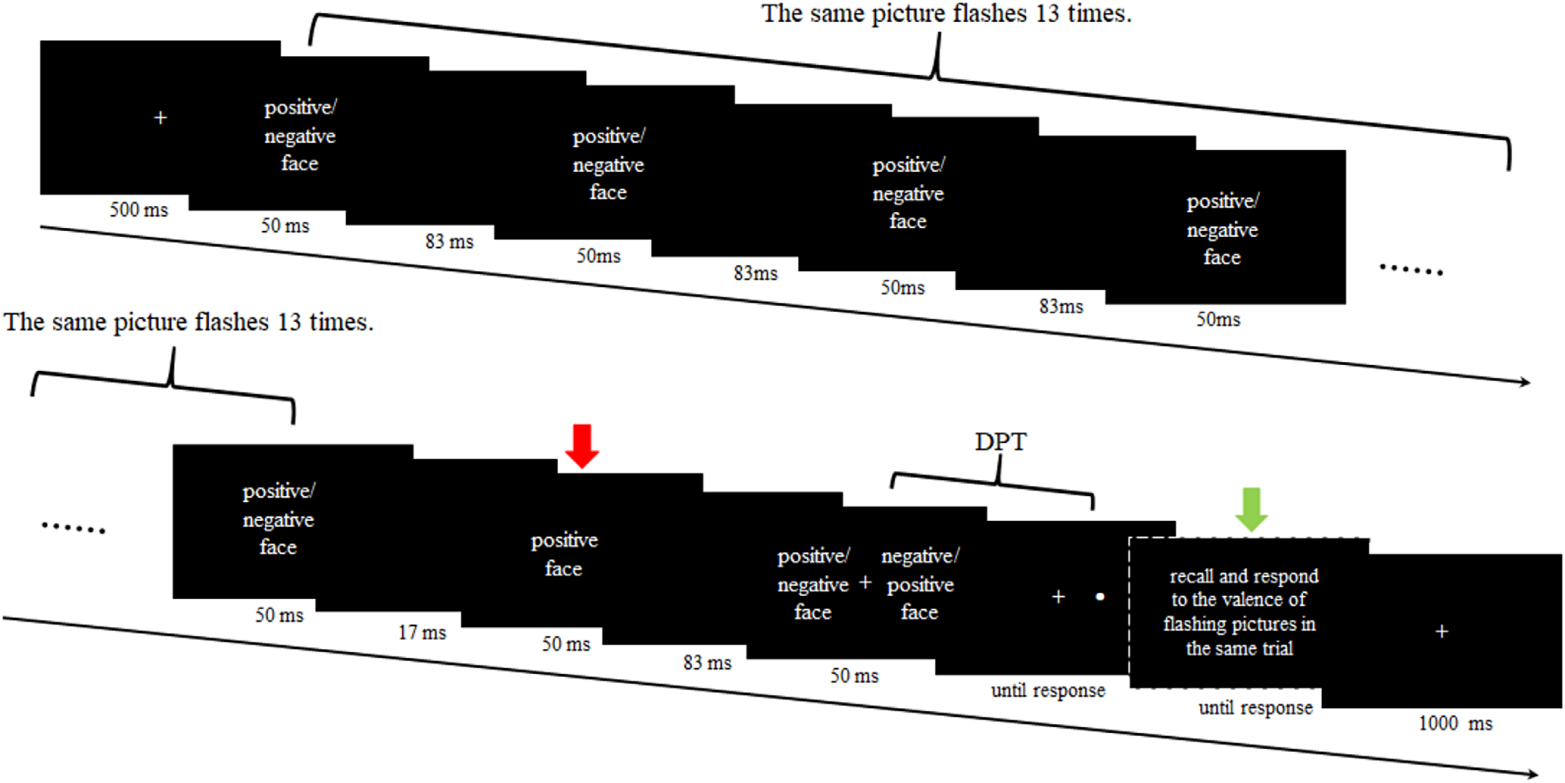
The illustration of the rhythmic training task. The same picture flashed 13 times to induce a stable theta rhythm. The screen highlighted with the red arrow is the predicted theta trough, which only displays positive faces. The screen with the green arrow presents a valence‐judgment task, which is randomly presented 20 times out of 256 trials. Participants were asked to judge the valance (happy or sad) of flashing pictures to ensure that they paid attention to the flashing pictures.

The formal training program consisted of 256 trials. Every trial involved four consecutive stages. First, at the beginning of each trial, a fixation “+” was presented for 500 ms, followed by a happy or sad face for 50 ms, and then a blank screen for 83 ms. The same sequence of a 50 ms face followed by an 83 ms blank screen was repeated 13 times, except for the last time when the blank screen was only 17 ms. The 13 face pictures were the same one. The objective of this stage was to evoke the theta oscillation of 7.5 Hz (1000 ms/(50 + 83 ms)) corresponding to a 60 Hz screen refresh rate. Second, following this sequence, a happy face of the same actor was displayed for 50 ms, followed by a blank screen for 83 ms. The stimulus onset asynchrony (SOA) between the last picture and this picture was 67 ms (half cycle), leading to the two pictures being in opposite phases of the theta wave. Third, a pair of happy and sad faces of the same actor was presented for 50 ms, followed by a probe dot “●” at either position of the two pictures. Only the happy‐sad pair was utilized in this stage. Participants were asked to press the “F” key if the probe dot was on the left and the “J” key if the dot was on the right, as quickly and accurately as possible. The probe dot appeared with equal probability at happy and sad face locations, similar to the operation in the traditional DPT, which aims to purely explore the facilitating effect of theta troughs on attentional shifts while excluding the impact of the probability of connecting probe and happy face. Fourth, participants were required to recall the valence of the flashing picture, pressing the “↑” key if it was a happy face and the “↓” key if it was a sad face. After that, a blank screen was displayed for 1000 ms, followed by the next trial (see Figure 2). The valence‐judgment task was conducted to ensure the subject maintained perceptual sensitivity to the flickering face, positioning it at the peak of the theta wave and to place the subsequent happy face at the trough of the theta wave, thereby encouraging attention to be switched to the positive stimulus. Participants were asked to practice 16 trials, with 3 trials containing the valence‐judgment task, to be familiar with the rules before formal training.

### 2.6 Data Analysis

According to the components theory of AB, six attentional scores can be calculated from the responses of participants in the DPT under different conditions [24]. These scores were

$$\mathrm{NAB}\;\text{score}=p\left[N\right]\&S—\mathrm{pN}\&\left[S\right]$$

$$\text{Positive attentional bias}\;\left(\mathrm{PAB}\right)\;\text{score}=p\left[N\right]\&H—\mathrm{pN}\&\left[H\right]$$

$$\text{Negative facilitated attention}\;\left(\mathrm{NFA}\right)\;\text{score}=p\left[N\right]\&N—p\left[S\right]\&N$$

$$\text{Positive facilitated attention}\;\left(\mathrm{PFA}\right)\;\text{score}=p\left[N\right]\&N—p\left[H\right]\&N$$

$$\text{Negative difficulty in disengaging}\;\left(\mathrm{NDD}\right)\;\text{score}=p\left[N\right]\&S—p\left[N\right]\&N$$

$$\text{Positive difficulty in disengaging}\;\left(\mathrm{PDD}\right)\;\text{score}=p\left[N\right]\&H—p\left[N\right]\&N$$

where p = probe, N = neutral face, S = sad face, and H = happy face. The abbreviation p[N]&S stands for the mean reaction time (RT) when the probe is located in the position of a neutral face in the neutral‐sad pair, while pN&[S] for the mean RT, when the probe is located in the position of a sad face in the neutral‐sad pair. Other abbreviations adhere to the same regulation.

A bias score greater than zero indicates that a sad or happy face captures attention more readily than a neutral face. A facilitated score greater than zero suggests that a sad or happy face is more likely to attract attention than a neutral face. A disengagement score greater than zero indicates that it takes longer to disengage attention from a sad or happy face compared to a neutral face.

Following the Shapiro–Wilk normality test applied to the data, we found that some data did not follow a normal distribution. To address this, we added 50 to the original values of the dependent variable, converting all data to positive numbers. Subsequently, we employed a generalized linear mixed model (GLMM) with a Gamma distribution as the link function to examine the effects of time and group on participants' attentional scores. The analysis was conducted using R 4.3.3, and the glmer function from the lme4 package [25] was used to construct the GLMM. Time, group, and their interaction were included as independent variables, while individual differences among participants were treated as a random effect. Moreover, given the non‐normal distribution of the training data, this study still employed a GLMM to assess the effects of the valence of the flashing images used to induce theta rhythms (referred to as “rhythm's valence”), the valence of the stimuli at the probe dot location in the DPT (referred to as “probe dot's valence”), and the group as fixed effects, with individual differences among participants included as random effects, on the outcome of the DPT in the training procedure.

The relationships between the baselines of AB and its subcomponents and the variations of pre‐ and post‐training were assessed with a linear regression model constructed by scikit‐learn in Python. The training of the model was carried out with the following steps:

- Load standardized data and divide it into two parts for training and testing with a ratio of 7:3.
- Load linear regression model.
- Train the model with the training set.
- Test the model with the testing set.
- Calculate the coefficients, intercepts, R‐squares, mean squared error (MSE), and cross‐validation scores in different regression models.

## 3 Results

### 3.1 Participant Characteristics

General information of the sample at the baseline assessment was presented in Table 2. The only distinction between groups with and without NAB was in their NAB scores.

**TABLE 2:** Baseline characteristics of study samples.

Variable	Group without NAB (*n* = 25)	Group with NAB (*n* = 32)	Statistic
Age in years, *M* (SD)	19.80 (1.32)	19.78 (1.10)	*t* (55) = 0.06
Sex, *N* (%)			*χ* ^*2*^ (1) = 0.84
Male	14 (56.00)	14 (43.80)	
Female	11 (44.00)	18 (56.20)	
PHQ‐9, *M* (SD)	7.76 (5.65)	8.75 (4.40)	*t* (55) = −0.75
Negative AB, *M* (SD)	−6.46 (4.75)	8.37 (8.30)	*t* (55) = −7.96***

*Note:* ***p < 0.001.

Abbreviations: NAB, negative attentional bias; *M*, mean; SD, standard deviation; PHQ‐9, patient health questionnaires‐9.

### 3.2 Primary Outcome

#### 3.2.1 Attentional Scores

A decrease in NAB scores after training indicates that the training was effective compared with pre‐training. Based on this criterion, the effectiveness rate for the group without NAB was 16%, while the effectiveness rate for the group with NAB was 78%.

As shown in the GLMM results presented in Table 3, after accounting for individual differences as a random effect, the interaction between group and time was significant for NAB, NFA, NDD, and PAB. This suggests that the training exerted differential effects on these scores across the two groups.

**TABLE 3:** GLMM of attentional scores with time and group effects.

	Fixed effects	*B*	SE	*t*	Random effects	Var	SD
NAB	Intercept	3.73	0.04	95.00***	Sub	0.01	0.08
	Time	0.36	0.04	8.01***			
	Group	0.22	0.06	4.01***			
	Time × group	−0.53	0.06	−8.53***			
NFA	Intercept	3.81	0.04	96.75***	Sub	0.01	0.07
	Time	0.24	0.05	5.22***			
	Group	0.13	0.06	2.37*			
	Time × group	−0.36	0.06	−5.54***			
NDD	Intercept	3.84	0.04	98.26***	Sub	0.01	0.09
	Time	0.11	0.04	2.84***			
	Group	0.08	0.06	1.47			
	Time × group	−0.17	0.06	−2.95***			
PAB	Intercept	3.93	0.05	80.22***	Sub	0.01	0.12
	Time	−0.09	0.05	−1.83			
	Group	−0.18	0.07	−2.63**			
	Time × group	0.28	0.07	4.17***			
PFA	Intercept	3.92	0.05	81.47***	Sub	0.01	0.09
	Time	−0.02	0.05	−0.35			
	Group	−0.11	0.07	−1.63			
	Time × group	0.13	0.08	1.75			
PDD	Intercept	3.91	0.05	79.61***	Sub	0.01	0.11
	Time	−0.07	0.05	−1.25			
	Group	−0.06	0.07	−0.84			
	Time × group	0.13	0.07	1.75			

*Note:* *p < 0.05, **p < 0.01, ***p < 0.001.

Abbreviations: B, beta coefficient (estimate); NAB, negative attentional bias; NDD, negative difficulty in disengaging; NFA, negative facilitated attention; PAB, positive attentional bias; PDD, positive difficulty in disengaging; PFA, positive facilitated attention; SD, standard deviation; SE, standard error; sub, subject; Var, variance.

**FIGURE 3:**
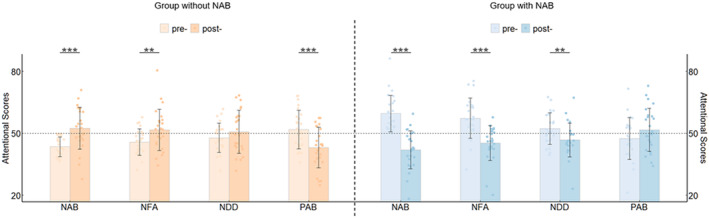
The training effect on NAB, NFA, NDD, and PAB for the group without NAB and the group with NAB, respectively. NAB, negative attentional bias; NFA, negative facilitated attention; NDD, negative difficulty in disengaging; PAB, positive attentional bias. **p < 0.01, ***p < 0.001.

Further analysis revealed that the NAB, NFA, and NDD scores decreased in the post‐test compared to the pre‐test in the group with NAB. For the group without NAB, the NAB and NFA scores increased in the post‐test relative to the pre‐test, whereas the PAB score decreased (see Figure 3).

A one‐sample *t*‐test was conducted for normally distributed data against a theoretical mean of 0, while a Wilcoxon signed‐rank test was used for non‐normally distributed data against a corrected theoretical mean of 50. The results indicated that only the NAB scores in the group with NAB were significantly higher than 50 before training and significantly lower than 50 after training, suggesting that the training effectively corrected the NAB (see Table 4).

**TABLE 4:** One‐sample *t* test or Wilcoxon signed‐rank test on attentional scores.

	*M* (SD)		Single‐sample *t* test		Wilcoxon test	
	Pre‐	Post‐	Pre‐	Post‐	Pre‐	Post‐
*Group without NAB*						
NAB	43.54 (4.75)	52.44 (10.12)	—	—	0.00***	210.00
NFA	−4.28 (6.36)	1.68 (10.02)	−3.37**	0.84	—	—
NDD	−2.18 (7.05)	0.76 (10.40)	−1.55	0.37	—	—
PAB	1.85 (9.35)	−6.93 (9.79)	0.99	−3.54**	—	—
PFA	1.16 (10.31)	−4.53 (8.43)	0.56	−2.69**	—	—
PDD	0.69 (9.41)	−2.14 (11.47)	0.37	−0.93	—	—
*Group with NAB*						
NAB	59.56 (8.79)	42.05 (9.23)	—	—	325.00***	35.00***
NFA	57.34 (9.78)	45.26 (8.39)	—	—	278.00***	61.50
NDD	52.22 (7.63)	46.78 (8.16)	—	—	205.00	83.00*
PAB	−2.51 (10.17)	1.67 (10.48)	−1.24	0.80	—	—
PFA	0.09 (11.91)	0.84 (10.55)	0.04	0.40	—	—
PDD	47.40 (9.59)	50.83 (12.53)	—	—	110.00	163.00

*Note:* *p < 0.05, **p < 0.01, ***p < 0.001.

Abbreviations: M, mean; NAB, negative attentional bias; NDD, negative difficulty in disengaging; NFA, negative facilitated attention; PAB, positive attentional bias; PDD, positive difficulty in disengaging; PFA, positive facilitated attention; SD, standard deviation.

#### 3.2.2 DPT In Training Procedure

**FIGURE 4:**
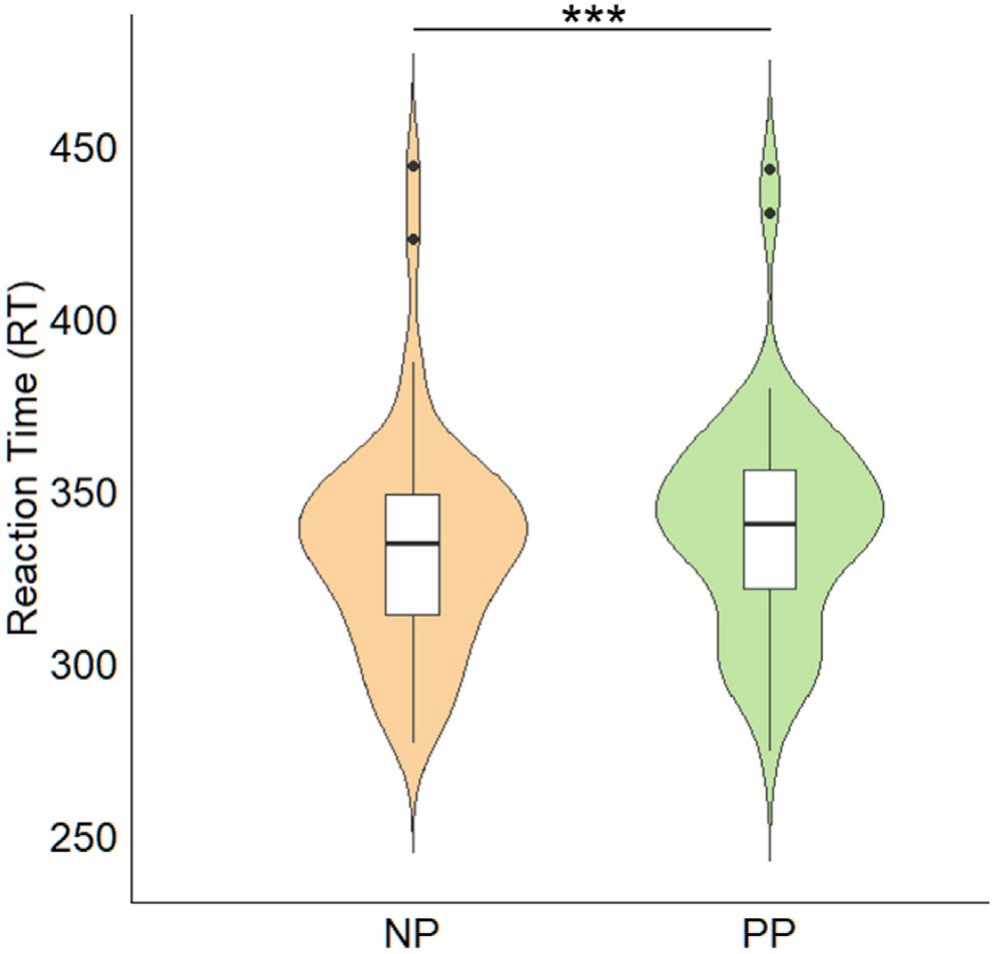
Simple effects analysis of rhythm's valence and probe dot's valence. NP means rhythm's valence was negative and probe dot's valence was positive; PP means rhythm's valence was positive and probe dot's valence was positive. ***p < 0.001.

As shown in Table 5, the main effect of the probe dot's valence was significant. More importantly, there was a significant interaction between the rhythm's valence and the probe dot's valence. Further analysis of simple effects (see Figure 4) revealed that when the probe dot's valence was positive, flashing negative stimuli more effectively facilitated participants' responses to the target compared to flashing positive stimuli (*M*_NP_ ± *SD* = 333.92 ± 4.56, *M*_PP_ ± *SD* = 340.01 ± 4.56; *z* = −5.17, *p* < 0.001).

**TABLE 5:** GLMM of DPT with group, rhythm's valence, and probe dot's valence effects.

Fixed effects	*B*	SE	*t*	Random effects	Var	SD
Intercept	5.81	0.03	226.21***	Sub	0.00	0.05
Group	0.01	0.04	0.28			
Rhythm's valence	0.01	0.01	1.05			
Probe dot's valence	−0.01	0.01	−2.18*			
Group × rhythm's valence	0.01	0.01	0.80			
Group × probe dot's valence	0.01	0.01	0.74			
Rhythm's valence × probe dot's valence	0.02	0.01	2.16*			
Group × rhythm's valence × probe dot's valence	−0.01	0.01	−1.02			

*Note:* *p < 0.05, **p < 0.01, ***p < 0.001.

Abbreviations: B, beta coefficient (estimate); SE, standard error; SD, standard deviation; sub, subject; Var, variance.

### 3.3 Exploratory Outcome

**FIGURE 5:**
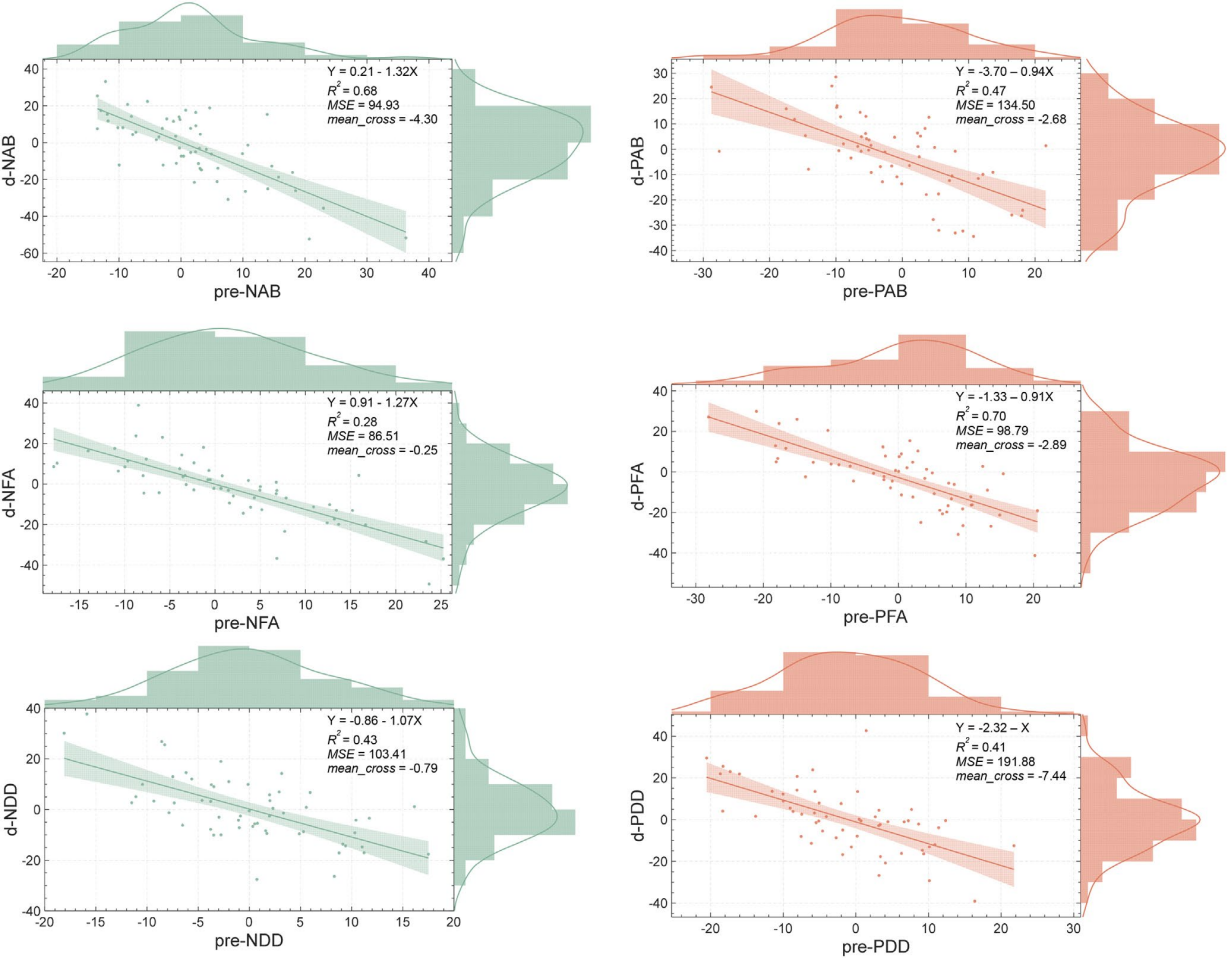
The baselines of attentional bias and its subcomponents predicted the amount of change by training. d‐NAB means the change in negative attentional scores before and after training, calculated as the post‐test scores minus the pre‐test scores. Pre‐NAB means the baseline of negative attentional bias scores before training. Other abbreviations adhere to the same regulation. NFA, negative facilitated attention; NDD, negative difficulty in disengaging; PAB, positive attentional bias; PFA, positive facilitated attention; PDD, positive difficulty in disengaging.

As shown in Figure 5, all baseline measures of AB and its subcomponents were predictive of the variations observed following the training. After training, all indicators showed a reduction, suggesting that the training facilitated the shift in attention, leading to a reversal of AB.

## 4 Discussion

### 4.1 Main Findings

The current study examined a new ABMT paradigm, combining theta rhythm with traditional DPT, and investigated whether the induced theta rhythm was effective in modifying the NAB. Following a single session of rhythmic training, individuals with initial biases exhibited a decrease in NAB scores compared with those without NAB before the training, which may be attributed to the beneficial effects of salient positive stimuli at the critical theta trough. The regression models indicated that the greater the initial negative bias, the larger the variation between pre‐ and post‐training and the more successful the training was.

Consistent with our hypothesis, individuals with an initial NAB were more likely to benefit from rhythm training. Hsu et al. [26] found that depressed individuals with NAB could gain from ABMT. In contrast, when participants exhibited no AB toward negative stimuli, the effect of ABMT was diminished, with attentional scores tending toward the median. In the novel paradigm that combines theta rhythm with DPT, the probability of the probe dot appearing at the positive stimulus location was 50%, for which previous studies have suggested that it might result in no training effect [13, 26]. Therefore, we attribute the training effect to the attentional shift induced by the trough of the theta rhythm. According to the rhythmic theory of attention [16], the trough of theta rhythm facilitates attention shifts. First, we evoke theta rhythm through neural entrainment [17]. Even after the rhythmic stimuli cease, entrained neural oscillations can persist for several cycles [27], allowing both the positive stimulus and the DPT to align with the theta trough. As theta oscillations commence, attention is directed toward the flashing images, and when the shift in attention occurs, participants encounter positive stimuli. The use of theta troughs to facilitate attention transfer forms the core mechanism of this training. The significant changes observed in NFA and PFA following training further support the hypothesis that the theta trough promotes attentional flexibility, primarily shifting attention from negative to positive stimuli.

The training procedure was designed to direct participants' attention toward positive stimuli. To achieve this, only positive images were presented at the critical theta rhythm trough. When negative images were used to induce theta rhythms, the positive image at the trough acted as a salient stimulus. Specifically, among the series of negative stimuli preceding the DPT in the training procedure, only the positive image at the final trough served as a feature singleton [28]. Salient stimuli are well known for effectively capturing attention [29], and previous studies have shown that responses are faster when the task target is a salient stimulus [30, 31], which was also observed in the present study. In contrast, when positive stimuli were repeatedly flashed, the consistency of the stimuli reduced participants' sensitivity to the positive image at the trough, leading to a diminished attentional effect and slower responses in the subsequent DPT. These findings suggest that future research may achieve more effective training outcomes by using negative stimuli to induce theta rhythms.

Given the practical relevance of the training, we focused primarily on negative attentional scores in individuals who exhibited NAB before training (i.e., pre‐test scores greater than zero) and on positive attentional scores in individuals who lacked PAB at baseline (i.e., pre‐test scores less than zero). The baselines of all attentional scores were found to negatively predict changes in corresponding attentional performance. This finding is consistent with both intuitive expectations and prior research. For instance, Fox et al. [32] demonstrated that active ABMT had a greater impact on participants with an initial bias toward spider images. Additionally, research on ABMT and depression showed that the group is most likely to benefit from ABMT comprised of individuals with elevated depression and at least a moderate level of NAB [26]. In other words, the effectiveness of training is influenced not only by the properties of the training itself but also by the initial state of the participants, highlighting the importance of state dependence [33, 34].

### 4.2 Limitation

This study has several limitations that warrant consideration. First, due to the challenge of evaluating depressive symptoms repeatedly over a short period, the effects of this new paradigm on these symptoms could not be assessed. Second, in the absence of a comparison with traditional DPT training, it remains unclear whether this approach is more effective than conventional methods in reducing NAB. Third, this study involved only a single session of training, limiting our ability to evaluate the long‐term effects of training on reducing NAB. Future research should address these issues to further enhance the effectiveness of this new training paradigm.

## 5 Conclusions

The theta‐based DPT effectively corrects negative attentional bias, and its success is influenced by the trainee's pre‐training state. Unlike probability‐based methods, this approach modifies negative bias through rhythmic attention shifts, and it achieves a particularly enhanced effect when a salient positive stimulus is presented at the critical theta trough following a sequence of flashing negative stimuli. Overall, this training task introduces a novel framework for attentional bias intervention.

## Author Contributions

G.L.: investigation and writing original draft. X.C.: review and editing. Y.W.: conceptualization and supervision. All authors contributed to the article and approved the submitted version.

## Ethics Statement

The authors assert that all procedures contributing to this work comply with the ethical standards of the relevant national and institutional committees on human experimentation and with the Helsinki Declaration of 1975, as revised in 2008.

## Conflicts of Interest

The authors declare no conflicts of interest.

## Data Availability

The data that support the findings of this study are available from the corresponding author upon reasonable request.
